# Clinical supervisors’ experience of a first-time application of entrustable professional activities in clinical supervision of medical students: findings from a Swedish pilot study

**DOI:** 10.1186/s12909-024-05211-w

**Published:** 2024-03-15

**Authors:** Paul Pålsson, Anna Cederborg, Monica Johansson, Helena Vallo Hult, Silvana Naredi, Katarina Jood

**Affiliations:** 1 Department of Education, Region Västra Götaland, NU-hospital group, 46185 Trollhättan, Sweden; 2 Institute of Neuroscience and Physiology, Department of Clinical Neuroscience, Sahlgrenska Academy, University of Gothenburg, Gothenburg, Sweden; 3 Institute of Medicine, Department of Molecular and Clinical Medicine, the Sahlgrenska Academy, University of Gothenburg, Gothenburg, Sweden; 4 Region Västra Götaland, Department of Internal Medicine, Sahlgrenska University Hospital, Gothenburg, Sweden; 5 Department of Education and Special Education, University of Gothenburg, Gothenburg, Sweden; 6 School of Business, Economics and IT, Department of Informatics, University West, Trollhättan, Sweden; 7 Institute of Clinical Sciences, Department of Anaesthesiology and Intensive Care, Sahlgrenska Academy, University of Gothenburg, Gothenburg, Sweden; 8 Region Västra Götaland, Department of Neurology, Sahlgrenska University Hospital, Gothenburg, Sweden

**Keywords:** Entrustable Professional activities, Clinical supervisor, Supervision, Medical student, Faculty development

## Abstract

**Background:**

Recently, all medical universities in Sweden jointly developed a framework for Entrustable Professional Activities (EPAs) for work-based training and assessment. This framework is now being introduced nationally in the new 6-year undergraduate medical programme that directly lead to a licence to practise. When EPAs are introduced, it is of central importance to gain clinical supervisors’ acceptance to apply the framework in their supervision of students. The aim of this study was therefore to investigate how clinical supervisors, not familiar with EPAs, experience clinical supervision using the framework for EPAs.

**Methods:**

We used a purposive sampling to recruit clinical supervisors. They were given written information on EPAs with a selection of suitable EPAs and the Swedish observation rating scale for assessment of autonomy, and they were offered to attend a 30-minute introductory web course. The participants were informed that EPAs were to be tested, and the students were asked to participate. After the study period the clinical supervisors participated in semi-structured interviews. Inductive qualitative content analysis was used to analyse the transcribed interviews.

**Results:**

Three general themes emerged in the qualitative analysis: Promoting Feedback, Trusting Assessments and Engaging Stakeholders. The participants described benefits from using EPAs, but pointed out a need for preparation and adaptation to facilitate implementation. The structure was perceived to provide structured support for feedback, student involvement, entrustment decisions, enabling supervisors to allow the students to do more things independently, although some expressed caution to rely on others’ assessments. Another concern was whether assessments of EPAs would be perceived as a form of examination, steeling focus from formative feedback. To understand the concept of EPA, the short web-based course and written information was regarded as sufficient. However, concern was expressed whether EPA could be applied by all clinical supervisors. Involvement and adaption of the workplace was pointed out as important since more frequent observation and feedback, with documentation requirements, increase the time required for supervision.

**Conclusions:**

EPAs were accepted as beneficial, promoting structured feedback and assessments of the students’ autonomy. Preparation of supervisors and students as well as involvement and adaptation of the workplace was pointed out as important.

## Introduction

Entrustable professional activities (EPAs) were introduced as a concept in competency-based medical education in 2005, and was developed as a mean to translate competencies into clinical practise [1–3]. EPAs can be defined as observable, measurable work-based activities that can be entrusted to a competent student or doctor in training [4]. While competences are attributes that the learner can have, EPAs consist of units of work that the learner can perform [2]. EPAs were first introduced in postgraduate medical education but over time, the concept has increasingly been applied to undergraduate medical education [5–7].

Medical education in Sweden is currently undergoing changes. In 2019, the Swedish Government decided to replace a 5.5-year undergraduate programme followed by a required 1.5 years of internship before applying for a licence, with a 6-year undergraduate programme that directly enables application for a licence to practice. To ensure that each graduate will reach the expected competence and be ready to perform clinical tasks central for the care of patients, the seven medical universities in Sweden jointly decided to introduce EPAs as a national framework for work-based assessments. In a national collaboration, using a modified Delphi procedure involving teachers from all Swedish medical schools, they defined ten core EPAs (Table 1) and developed an observational entrustment-supervision scale for the Swedish undergraduate medical education (Table 2) [8].

**Table 1:** Swedish core entrustable professional activities (EPAs) for undergraduate education

1* Gather a history and perform a relevant physical examination
2* Prioritize a preliminary diagnosis among relevant differential diagnoses
3* Formulate an initial plan for investigations
4* Formulate and implement an initial management plan
5 Identify the need for and initiate interventions to promote health and prevent illness
6 Perform general procedures of a physician
7 Recognize patients requiring urgent care and initiate primary intervention
8* Summarize, document, prescribe and issue medical certificate based on a patient encounter
9* Collaborate within healthcare and with other professionals in the community
10 Contribute to a patient safety culture within healthcare

*EPAs suggested in the written information to the clinical supervisors

**Table 2:** The Swedish retrospective observation rating scale for undergraduate education

Description
The student was an active observer while I did the activity
The student did the activity together with me
The student did the activity, I was there and had to intervene or prompt
The student did the activity, I was there and did not need to intervene or prompt
The student did the activity, I was nearby and had to intervene or prompt
The student did the activity, I was nearby and did not need to intervene or prompt

The scale is used together with an open-ended question for feedforward

Five levels of autonomy in performance of EPAs have been described and incorporated in supervision scales as tools for assessment of EPAs in clinical practise [4, 9, 10]. The first level is that the student observes the supervisor when performing the task, the second level is that the student performs the task together with the supervisor, the third level is that the student performs the task with the supervisor available if needed, the fourth level is that the student performs the task independently and the fifth and final level is that the student is able to teach the task However, in undergraduate medical education most EPAs will not be possible to entrust students to perform independently or to teach. Level four and five are therefore not included in the Swedish observation rating scale for undergraduate medical education [8]. Moreover, a retrospective/ co-activity design for the rating scale was chosen (Table 2), meaning that the scale is based on the degree to which the supervisor had to intervene when a student performed an EPA [8, 11].

This framework for EPA is now being implemented as a general tool for work-based training and assessment for the new 6-year medical programme. In this level, the clinical supervisors play a central role, as their acceptance and application of the framework is of paramount importance for the concept. The clinical supervisors are expected to demonstrate to the students how the tasks that constitute chosen activities in the EPA framework should be performed, to give formative feedback when the student performs the tasks, and finally, to assess the level of supervision that the student needs.

To date EPAs have scarcely been used in post graduate medical education in Sweden, meaning that most supervisors are not familiar with the concept. However, the clinical tasks defined by EPAs are expected to be well-known by the clinical supervisors as they represent common tasks performed in every day clinical practise. Moreover, making decisions on how much supervision a student needs are the not new for clinical supervisors, as ad-hoc entrustment decisions occur in clinical every day practise when a learner at any level is supervised [12]. As the student’s competence grows more and more, autonomy can gradually and safely be permitted by the supervisor. Despite this, applying the framework of EPAs in clinical supervision of medical students will constitute a new task for the supervisors. Within their daily clinical practise, they are expected to involve students and allow them to practise EPAs in authentic situations, at appropriate levels of autonomy, according to the individual level of competence, as well as to provide purposeful feedback and feedforward. Thus, when implementing the framework for EPAs, in order to safeguard its application, the faculties must provide a proper introduction for the supervisors. However, empirical studies reporting how supervisors perceive and integrate EPAs for clinical supervision in their daily practise are scarce [13–15]. Enhanced knowledge in this area is important, as it may facilitate the development of adequate training and support to supervisors with no experience of EPAs in performing their task, ultimately translating into improved learning possibilities for students.

Therefore, the objective of this paper was to investigate how clinical supervisors, not familiar with EPAs, perceive clinical supervision using the EPA framework. For this purpose, we conducted this pilot study and applied a qualitative research method.

## Methods

### Setting

This study was performed in Region Västra Götaland in the south-west of Sweden, a region with 1.7 million inhabitants. The University of Gothenburg within this region has a medical school. An agreement between the Swedish state, the region, and Gothenburg university, requires all health care facilities within the region to assist with clinical supervision for medical students. Therefore, medical students complete clinical rotations both at the university hospital and at non-university hospitals in the region.

### Respondents and recruitment

Clinical supervisors from different hospitals in the region were invited. They received a short web-based briefing of EPAs and then tried the EPA concept in their clinical supervision. The supervisors used the Swedish core EPAs (Table 1) and the Swedish retrospective observation rating scale for undergraduate education (Table 2).

To ensure diversity among study respondents, different clinical rotations throughout the medical programme were selected for inclusion, including different disciplines and different types of hospitals. Clinical supervisors working at the same department as the interviewer were not invited to participate to avoid personal relations influencing the results. The contact persons responsible for medical students at the selected departments were asked to recruit participants with different levels of experience. Participants were included as they agreed to participate.

Respondents received written information and gave informed consent for the study before taking part in study procedures. The information included the purpose of the study, study procedures, a short-written introduction to EPAs as well as an invitation to a 30-minute introductory web-course on how to use EPAs for supervision and assessment in clinical practice [16]. The web course included an introduction to the Swedish core EPAs and the Swedish retrospective observation rating scale for undergraduate education. The course covered the characteristics of EPAs and gave examples on how they can be used in clinical supervision, as well as instructions on how to give useful feedback, including practical tips based on an article by Ramani et al., such as giving feedback timely and individually, starting with the student’s reflection, acknowledging what went well as what the student needs to correct, and concluding with an action plan for further development [17]. The supervisors were given a list of suitable EPAs selected from the Swedish Core EPAs for undergraduate education (Table 1). During the study period, the participating supervisors informed the students they supervised at the ongoing 5.5-year medical program, that they were testing the use of the EPA concept for supervision and asked the students to volunteer to test the new teaching method. The medical students did not receive any additional introduction to the EPA concept, and as the concept was not yet implemented formally, a procedure for entrustment decisions by an examiner or a entrustment committee based on collected ad-hoc assessments had not been established.

### Data collection

Prior to the interview, the authors developed a semi-structured interview guide. The guide intended to explore the respondents’ experiences using EPAs in their clinical practice, as well as their reflections on how EPAs can be introduced in their teaching setting, see appendix 1. The interview guide was pilot tested in the first two interviews. As these pilot tests resulted only in minor revisions of the interview guide, the first two interviews were included in the analysis. All interviews were conducted by the first author, using Microsoft Teams [18] from 11th November 2022 to 6th March 2023. The interviews were held in a conversational style, following the interview guide. Probing questions were used to further explore and clarify the responses from the respondents. The interviews were recorded using recording feature of Microsoft Teams and lasted in median for 34 min (25–49 min). In the last two interviews no new concepts emerged, the research group then concluded that data saturation was reached.

### Analysis

The recordings of the interviews were transcribed verbatim. After transcription, the recordings were deleted, and the transcripts were anonymised. The participants were not invited to comment on the transcripts. Data analysis was done after all interviews were transcribed. All authors familiarized themselves with the manifest data, by thoroughly reading all the transcripts and individually coding the material. Inductive, qualitative content analysis was conducted using the methodology described by Graneheim [14, 15], using the Nvivo 12 software [16]. Quotes were selected from the text, condensed as meaningful units and labelled with codes. The codes were sorted into sub-themes in an iterative process in a series of meetings within the author group to identify subthemes. Finally, the latent content was used to generate the main themes in the material.

## Results

Ten clinical supervisors were recruited. The supervisors’ clinical experience ranged from interns and residents with less than 5 years of clinical experience, to senior consultants with over 25 years in clinical practice (Table 3). All respondents had previous experience in clinical supervision of medical students, but no one had any experience with using the EPA framework.

**Table 3:** Respondent characteristics

Characteristics	N
*Clinical competence level*	
Intern	2
Resident	4
Consultant	4
*Hospital type*	
University hospital	2
Non-university hospital	8
*Gender*	
Female	4
Male	6

All clinical supervisors reported that they had been able to supervise and give feedback using EPAs. The most frequently used EPAs were gathering a patient’s history and performing physical examinations. In general, the supervisors found the model useful and beneficial to the students’ learning.

Three general themes emerged in the qualitative analysis: Promoting Feedback, Quantifying Autonomy and Engaging stakeholders. Each theme had three subthemes. For a list of themes, subthemes and example quotes, see Table 4.

**Table 4:** Themes, subthemes and example quotes

Themes	Sub-theme	Example quote
**Theme 1: Promoting Feedback**	**Using EPA requires the supervisor to give feedback**	*It says, for example, about what the student can do to increase their independence and then about the student having a plan how to achieve that. The possibility that it can help with concrete feedback on improvement and not just sort of saying “yes about this, it went really well”.* (R4)
	**Clear structure for feedback**	*After all, it is a template so that there will be an equal evaluation for everyone. That regardless of who you are, you do an evaluation in the same way and then it becomes clearer for the candidates as well, that there is structure and clarity in being evaluated in the same way every time* (R7)
	**The student needs to actively seek feedback**	*If you have a form you have to fill out or an app you must submit to be approved, then the student is forced to search for more feedback and the supervisors are forced to give it, so I think it is an advantage to have all different kinds of these forms.* (R8)
**Theme 2: Trusting Assessments**	**Structured assessment of level of independence**	*It is also good to talk about it together so that both the student and the supervisor agree on what level of independence the student is at, so that it does not happen that a given task feels too difficult or too easy /…/ it is simply like a quality control, that the person views the goals, that it is not only theoretical, but that it is still our task with the clinical rotation to see that the practical parts also work.* (R2)
	**The observation rating scale as an assessment**	*If you look at several assessments from a more meta-perspective as an examiner or if you look at several assessments and combine them then it could be an advantage to have a scale, but in the individual situation, I probably think that what for me feels like the most important feedback is the one where we are actually talking about it and maybe not the actual number.* (R6)
	**Trusting the entrustability assessment**	*If the department calls about a patient whose circulation is failing, for example, you could then let them go initially, if they have assessed that type of patient before and if the students perform the activity themselves as level 5* [with the supervisor nearby], *then you could let them go and do that assessment themselves initially.* (R1)
**Theme 3: Engaging Stakeholders**	**Preparing the supervisor**	*It is enough that there is a piece of paper or a website with a list on it, it does not need to say more, the student must be able to perform a rectoscopy, the student must be able to insert a NG-tube.* (R9)
	**EPA as a tool for all clinical supervisors**	*If you just use EPA a lot then there is no difficulty in introducing it. Everyone who supervises students will be able to learn this and apply EPA and make decent judgments with some dispersal, but if you only have a student at the clinic once, or a student attending the clinic once per semester or every three months, then you will not be able to manage this.* (R9)
	**Teaching takes time**	*I also think that there is a challenge in that you actually take the time to find a space and go and actually give feedback on what you are doing if it is for example a clinical consultation or a patient examination.* (R3)

R = Respondent

### Theme 1: promoting feedback

#### Using EPA requires the supervisor to give feedback

The structured model for feedback integrated into the framework for EPAs was perceived as beneficial to the student. Several respondents described how the structure created an incentive for the supervisors to provide feedback on specific details that the students could improve. *“That there is a possibility to actually receive concrete feedback because it should be documented and there should be a requirement to receive feedback, and it shouldn’t just be: just keep on doing as you do.”* (Respondent 3) However, contrasting opinions surfaced as well. Although the general perception was that the feedback would be useful to the students, one respondent expressed concern that supervisors would use less useful standard phrases. For example, “keep up the good work”, or by just suggesting that the student should examine more patients to get more experience. *“What should the student do, continue to examine more”* (R2).

#### Clear structure for feedback

Using the same template to provide feedback to students in all teaching encounters was seen as beneficial for the students, as well as for the supervisors as both can have a clear expectation on how feedback will be given. *“For those who are used to working with it a lot, it will be a clear improvement because you will have a better structure of your assessments and your feedback.”* (R9) However, the issue was raised that different supervisors may have different standards when it comes to some activities, such as writing a medical record.

### The student needs to actively seek feedback

A returning theme was how the EPA framework encouraged students to reflect and make plans for improvement. *“It became more the candidates themselves who reflected, and I could provide input based on their own reflection as well as what I had reflected on, so for me I thought it gave a good structure.”*(R7) The respondents described how involving the students increased the students self-awareness, and highlighted that the EPA framework encouraged them to actively seek feedback: *“Yes but it is like night and day from today, I think they become more included and understand their shortcomings and development possibilities and development potential and things like that because it is so concrete and it is so clear what they need to achieve and so on, so I think that their participation in the whole thing will also make them more understanding and understand that this is how I have to proceed.”*(R5).

### Theme 2: trusting assessments

#### Structured assessment of level of independence

Although several respondents described that they, to some extent, assess the level of independence in their current practice, the more structured method used with EPAs, including clear levels, was seen as beneficial to the students. *“I think EPA is good, it is a structured way to follow the candidates’ development and it is also a concrete tool for them to know how they are doing.”* (R5) One respondent described how the observation rating scale provided a tool to follow the student’s development as the student acquired a new clinical skill: *“For example, I had a student who observed six rectoscopies, and through that I could see his development. At first, he simply observed while I performed the procedure, then he had the opportunity to examine after I inserted the instrument. In the last two cases, he performed the examination himself while I observed and provided feedback afterwards.”* (R 9).

Some concern was raised when it came to the level of inter-observer variability in the assessment. In such cases the respondents pointed out a concern that different supervisors might assess the student’s level of independence differently. *“We are all unique individuals who come to assess them, so naturally, I may have an opinion on something while others might not have any thoughts about writing a journal entry or an admission note. I feel that something is amiss, that they need to add something, while others argue that no, what’s already there is entirely adequate. It’s challenging to determine, as it always turns into a personal reflection on one’s usual approach.”* (R10).

#### The observation rating scale as an assessment

In general, the observation rating scale was perceived as a tool to evaluate the students over time, especially after a number of observations. It was seen especially useful for the main clinical supervisors. *“For me specifically, as someone who is in charge and responsible, EPA is indeed a great tool to track the candidate’s progress. It allows me to identify areas that may pose challenges and say, “It seems like this is a bit tricky, come with me and let’s do some extra practice,” even if I am not present on the same ward or department as the student.”* (R5) Several respondents raised concern that attributing a number on the observation rating scale would be seen as summative assessment, and that it, as such would be perceived as a form of judgement or examination by the students, steeling focus from the formative feedback. *“I think that the students, unfortunately, like in all examinations, focus more on the grade they get, i.e. the level assessment, which will perhaps cloud their memory of what was said and it will be good if you document it so that they can read in their little track app whatever it is you have told them.”* (R9).

#### Trusting the entrustability assessment

There was a general perception that the observation rating scale was useful, and would enable supervisors to allow the students to do more things independently. *“It is probably the advantage of the other colleagues having assessed what this candidate is like in this observation scale that makes me feel more secure in perhaps letting them go a little bit.” (R5)* The respondents described that assessment of entrustability is already taking place, without the formal structure of EPAs as one respondent describes talking about use of the observation rating scale: *“Yes, like when I’m going to teach them to insert an IV, then I always ask before what level are you at, have you done it before and then you stand next to them and observe them so I don’t know if there really would be that much of a difference.”* (R1) In contrast one respondent expressed caution to rely on other colleagues’ assessments of the student’s entrustment level. *“If I have never met the student before, I would never dare to start at a Level 5 or 6* [with the supervisor nearby]. *An unfamiliar student would never be allowed to conduct a patient conversation without me being present in the room, listening to what is being said and observing what is being done. I’m afraid I would constantly feel the need to intervene.”* (R9).

### Theme 3: engaging stakeholders

#### Preparing the supervisor

There were several suggestions for ways to prepare the supervisors to use EPAs. They ranged from handing out small pocket cards explaining the model, to shadowing an experienced supervisor, putting up posters on the ward walls with information, and creating websites with course-specific information (Table 5). All respondents reported that the digital 30-minute course they had attended was sufficient to prepare them for the general concept of EPAs. *“I think it is good to have an information course prior, like the online education course, just to learn what an EPA is before the new concept arrives”* (R7) Several respondents pointed out a need for course specific details, as well as general information. *“I think the course leader actually can send out what they want to, what they think should be assessed, and I think there is an advantage in the students also knowing which EPAs they will be assessed on, so that you have some sort of clarity.”* (R3) Of note, one supervisor pointed out that supervisors new to EPAs could be instructed by the students. *“The uncertainty that I might feel and project onto my colleagues may not matter that much because [the students] will say, “Here, I want you to… How did this go for me? What is your email address, and what should I keep in mind?” and then they will solve it.”* (R4).

#### EPA as a tool for all clinical supervisors

Some respondents believed that it would be difficult to expect all physicians to supervise using the EPA framework. Instead, they suggested a strategy where a smaller group of supervisors would do all the clinical supervision. *“With a large group of teachers, I think it will be difficult to have a high level of quality on this. But if you decide that now it is the eight of us who will have the students this semester, then there are no problems at all.”* (R9) In contrast, others found the concepts simple to grasp, without much introduction. *“I do not think it is a complicated system, the observation scale, and this, that you assess independence, is very clear and easy to assess.”* (R1).

#### Teaching takes time

The clinical supervisors perceived supervision using EPAs to require more time, compared to how they were used to supervise. *“I’m concerned about the administrative burden it imposes on those primarily responsible for it, such as colleagues and junior doctors in the departments who don’t really have ample administrative time. I don’t know, it doesn’t take a lot of time, but there are many tasks that you’re asked to do that individually don’t take much time.”* (R4) *“It will take more time than it does now. /…/ we will not be able to do as much as we do now in our clinical everyday life, so much more will be required.”* (R10) However, several supervisors pointed out that supervising EPAs takes more time mainly because it requires the supervisor to actually supervise and give feedback. *“If you instead compare a structured model e.g. EPA compared to freebasing and guesstimate feedback, I think it can be more time-efficient to have a structure around feedback and especially if you give feedback in a way that the student is already used to and the supervisor is already used to, then you are both schooled on the structure and then it might go faster.”* (R6).

Several supervisors described a need for hospital management to recognize clinical supervision as an important objective of clinical work, alongside managing patients. “*There should be an acceptance [from management] that it actually takes time and that there is a consideration in the clinic as to why we are supervising them, why the students are there /…/ I miss a culture and an opportunity for supervision where we focus on why supervision is so important and why it is crucial to allocate time for it.”* (R3).

**Table 5:** Suggestions from respondents for the introduction of EPAs, provision of resources to support the supervisor, and how to adapt the clinical workplace

Domain	Suggestion
Introducing EPAs	• Web-courses on EPAs
	• Observing an experienced supervisor
	• Joint assessment with experienced supervisor
	• Written instructions on teaching objectives from faculty
	• Use the students as experts on EPAs
Providing resources to support the supervisor	• Website with course specific information on EPAs
	• Short video tutorials
	• Pocket cards with EPA-information
	• Posters with EPA-information in the workplace
Adapting the clinical workplace for supervision using EPAs	• Workplace EPA-meetings
	• Limit the number of physicians involved as EPA-supervisors
	• Dedicated time for teaching

## Discussion

This qualitative study aimed to explore how clinical supervisors new to the concept of EPAs, perceive the framework when it is introduced. The clinical supervisors described benefits for the students learning by using EPAs in their clinical supervision but pointed out a need for preparation and adaptation to facilitate implementation. Findings from this study provide contributions to three main aspects of previous research with implications for implementation of EPAs for clinical supervision.

First, our findings highlight that EPAs facilitate promoting feedback. Clinical supervisors described that EPAs required the supervisors to give more frequent feedback to the students. This is in line with previous research showing that the introduction of EPAs increased the frequency and quality of feedback, and motivated the students to request more feedback [19, 20]. However, the concern, expressed by some respondents, that some of the feedback tend be too vague to be useful for the students, is also supported by experience from others, reporting that a proportion of the written feedback on EPAs was deemed without guidance or actionable steps [21]. These results emphasizes the importance of providing supervisors with training in giving effective feedback when assessment of EPAs are implemented for clinical supervision [22].

Second, we found that entrustment decisions, previously seen as ad-hoc decisions, gained a coherent structure by using the entrustment scale. However, some supervisors were reluctant to trust other colleagues’ decisions on autonomy. The hesitancy to trust previous entrustment decisions is a recurring theme in previous research [23, 24]. Moreover, supervisors have been shown to be reluctant to fully trust tasks to the students [25]. Ten Cate et al. propose three types of trust; *presumptive trust*, based entirely on credentials or previous assessments, *initial trust*, based on first impressions and *grounded trust* based on prolonged experience with the trainee [26]. A range of factors have been shown to influence entrustment decisions, such as students characteristics, supervisors characteristics and the task at hand [14, 27]. Generally the students tend to assess their level of independence higher than the supervisors assessments [28–30]. For entrustment decisions to be useful for the students they need to result in an increased autonomy [31]. While assessments of EPAs by previous supervisors provide presumptive trust, there is a need to develop the supervisor-student relationship to form grounded trust, to ensure that the supervisor will allow the student to act with increased independence. If supervisors underestimate the students’ skills and do not entrust them to perform at their true level of independence in the care of patients, it may limit the learner’s development. On the other hand, overestimations can potentially harm patients [32]. Thus, a central challenge is to make the educational terminology and the concept of EPAs understandable and valid for the clinical supervisors. While the respondents in our pilot study used EPAs and an observation rating scale, the students were from a curriculum not applying EPAs. Therefore, there were no previous summative assessments of the students available to the supervisors, and no formal procedures for such decisions. It is possible that the supervisors who expressed concern about trusting colleague’s decisions would be more confident in allowing students to train at an increased level of independence if they had access to summative assessments and with a formal procedure for entrustment decisions in place.

Finally, this study identified multiple dimensions on how to prepare clinical supervisors to use EPAs in their teaching practice. The clinical supervisors found the short, written instruction and web-course on EPAs sufficient as an additional preparation to use EPAs in their clinical practice. However, they also pointed out the need for additional resources to better prepare them for their role. There is a well-known variation in how physicians supervise in clinical practice and to what extent their supervision is in line with pedagogical theory [33–35]. Physicians who get involved in the role as clinical supervisors need to understand theories for work-based learning and their role as teachers [36, 37]. Especially they need to know the students´ learning objectives and how to adjust their supervision to match the learners´ needs [38–41]. Our findings point out the need for the university to provide supervisor guides, co-mentoring with experienced supervisors and a course on EPA-supervision to prepare clinical supervisors. Previous research has identified the importance of preparing supervisors on how to use EPA, [13, 42] as well as a need for targeted faculty development [43]. The skills the supervisor need include techniques for observation and feedback and understanding of the curriculum [44, 45]. Short introductions paired with faculty guides and web-based EPA tool kits have been shown to be effective [46, 47]. Our results emphasize that while the supervisors perceived the structure for feedback as feasible and beneficial for the students, they expressed concerns with other aspects of the framework for EPAs. These concerns included inter observer variability of scale used for assessments, difficulties in providing quality feedback frequently, hesitancy to trust entrustment decisions made by others, and that formative assessments eventually will end up in summative assessments. Thus, our results points at the importance to specifically address these aspects when EPA are introduced to clinical supervisors.

In addition to resources provided by the university, we found a need to adapt the workplace to accommodate the supervisor role alongside the tasks as a physician. The challenge to adapt the clinical workplace, primarily focused on providing safe healthcare, to accommodate clinical supervision is well known [33, 48–50]. Lack of time to teach is frequently seen as a barrier to effective clinical supervision [19, 33, 34, 51–53].

### Study strengths and limitations

A strength of this study is that it involved clinical supervisors with different levels of experience, from several hospitals, ranging from a large university hospital to smaller local hospitals, in a setting where EPAs are not commonly used in medical education. It adds knowledge on how clinical supervisors new to the EPA-framework perceive its usefulness in their clinical teaching practice, and their viewpoints on how to prepare clinical supervisors to teach using EPAs. The results are limited to the context of one Swedish university with regionalised clinical rotations. However, the findings are in line with previous research on EPA-implementation in other settings. As for all research, the study has limitations. Despite our efforts to include participants with different backgrounds there is a risk that those that volunteer to participate have a greater interest in medical education and differ in viewpoint from the general physician population.

## Conclusion

The EPA framework was accepted as feasible and beneficial for the students by all clinical supervisors interviewed. The EPA concept promoted structured feedback to the student and the student had to actively seek feedback. Although some assessment of the level of independence is made by supervisors in current practice, EPAs were perceived to enable a more structured assessment of the level of independence. While respondents expressed that a short web-based course and written information was regarded as sufficient to understand the concept of EPAs, concern was expressed about whether all supervisors would be able to assess EPAs, and that assessments of EPAs would require more time for supervision.

## Data Availability

The datasets used and/or analysed during the current study available from the corresponding author on reasonable request.
